# Proven pulmonary aspergillosis in a COVID-19 patient: A case report

**DOI:** 10.18502/cmm.7.2.7031

**Published:** 2021-06

**Authors:** Sadegh Khodavaisy, Nasim Khajavirad, Seyed Jamal Hashemi, Alireza Izadi, Seyed Ali Dehghan Manshadi, Alireza Abdollahi, Amir Aliramezani, Elahe Sasani, Mahsa Abdorahimi, Reyhaneh Sadat Kiyaeie, Zohre Khosravany, Muhammad Ibrahim Getso, Mohammadreza Salehi

**Affiliations:** 1 Department of Medical Parasitology and Mycology, Faculty of Public Health, Tehran University of Medical Sciences, Tehran, Iran; 2 Department of Internal Medicine, Faculty of Medicine, Tehran University of Medical Sciences, Tehran, Iran; 3 Department of Infectious Diseases and Tropical Medicine, Imam Khomeini Hospital Complex, Tehran University of Medical Sciences, Tehran, Iran; 4 Department of Pathology, Imam Khomeini Hospital Complex, Tehran University of Medical Sciences, Tehran, Iran; 5 Central Laboratory, Imam Khomeini Hospital Complex, Tehran University of Medical Sciences, Tehran, Iran; 6 Department of Medical Mycology, Faculty of Medical Sciences, Tarbiat Modares University, Tehran, Iran; 7 Department of Microbiology, Shahr-e-Qods Branch, Islamic Azad University, Tehran, Iran; 8 Department of Medical Microbiology and Parasitology, College of Health Sciences, Faculty of Clinical Sciences, Bayero University, Kano, Nigeria

**Keywords:** Aspergillosis, *Aspergillus tubingensis*, COVID-19

## Abstract

**Background and Purpose:**

Coronavirus disease 2019 (COVID-19) has become a significant clinical challenge in healthcare settings all over the world. Critically ill COVID-19 patients with acute respiratory distress syndrome may be at increased risk of co-infection with pulmonary aspergillosis. This study aimed to describe a clinical case of proven pulmonary aspergillosis caused by *Aspergillus tubingensis* in a 59-year-old man with a history of hospitalization due to COVID-19 infection.

**Case report:**

The Covid-19 infection was confirmed by positive nasopharyngeal polymerase chain reaction. He had a cavitary lesion measured 20 mm in diameter with intracavitary soft tissue density in the left lung in the first chest computerized tomography scan. After 25 days, he showed two cavitary lesions in both lungs which raised suspicion of fungal infection; hence, the patient underwent a trans-thoracic biopsy of the cavitary lesion. The direct examination and culture of the biopsy material revealed *Aspergillus* species. To confirm the *Aspergillus* species identification, the beta-tubulin region was sequenced. The patient was treated with oral voriconazole.

**Conclusion:**

This report underlined the importance of early diagnosis and management of invasive fungal infections in severe COVID-19 patients.

## Introduction

The severe acute respiratory syndrome coronavirus 2 (SARS-CoV-2), responsible for the coronavirus disease 2019 (COVID-19) pandemic, is an emerging pathogen that has recently become a significant clinical challenge in healthcare settings all over the world [ 1 , 2 ]. The emergence of this disease was first reported in December 2019 in Wuhan city, Hubei province of China. It rapidly spread around the globe and became a pandemic threatening the whole world [ 1 ]. The disease presentions range from asymptomatic to severe lethal form [ 2 ] that includes severe respiratory disease associated with a high mortality rate that varies among countries [ 3 ]. Patients infected with the Coronavirus may experience severe damage to their lungs due to rapid replication of the virus, high cytokine secretions, and severe inflammatory reactions [ 4 ]. Unfortunately, severe lung damages expose patients to serious secondary infections, including fungal nosocomial infections [ 5 ]. 

*Aspergillus is* an opportunistic fungal pathogen that causes various diseases in immunocompromised individuals [ 6 ]. It should also be noted that patients with COVID-19 may be at increased risk of co-infection with pulmonary aspergillosis [ 4 ]. Invasive pulmonary aspergillosis (IPA) is a known complication of severe influenza pneumonia; it has been suggested that COVID-19 disease is a risk factor for the development of pulmonary aspergillosis infection [ 7 ]. Therefore, testing for the presence of *Aspergillus* species in respiratory secretions, biopsy, and bronchoalveolar lavage specimens of COVID-19 patients in the intensive care unit can be a positive step towards their better management and reduction of mortality in them. This case report presents a case of a COVID-19 patient with symptoms of pulmonary aspergillosis.

## Case report

A 59-year-old man with a history of hospitalization due to COVID-19 infection was referred to our ward three days after discharge on account of mild hemoptysis. He was a known hypertensive who underwent coronary artery bypass graft 3 years ago and was on aspirin, metoprolol, and losartan at the time of admission. He mentioned that he had had one episode of hemoptysis and coughed blood-streaked sputum a few times. The patient had an occupational history of working in a leather shoe factory for 32 years. 

**Figure 1:**
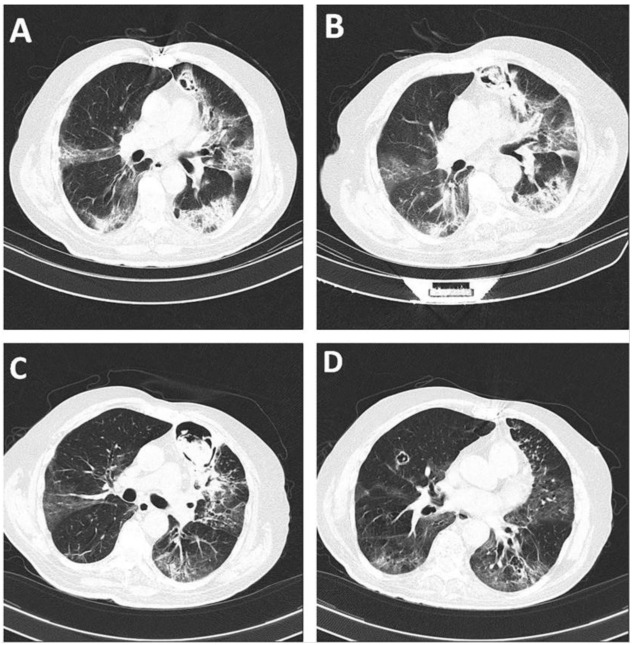
A) Chest computed tomography (CT) scan on the first day of the previous admission that indicates COVID-19 pneumonia and a cavitary lesion measured 20 mm in diameter. B) Chest CT scan on the 3rd week of the previous admission which shows an increase in cavity size. C) Chest CT scan on the second admission which shows a noticeable increase in the size of the previous cavity. D) Chest CT scan on the second admission that shows a new cavity in the opposite lung

As mentioned earlier, the patient had been previously hospitalized for one month due to COVID-19 infection confirmed by positive nasopharyngeal polymerase chain reaction. During the aforementioned hospitalization, his chest computerized tomography (CT) scan (performed on the first day of admission) had revealed ground-glass opacity and consolidation involving more than 40% of the pulmonary field (Figure 1. A). Besides, there had been a cavitary lesion measured 20 mm in diameter with intracavitary soft tissue density in the left lung. It is noteworthy that the results of his HIV and tuberculosis screening had been negative.

He had been treated with sofosbuvir/daclatasvir (for 10 days), interferon beta-1-a (five doses), and low-dose dexamethasone (8 mg/day for 5 days followed by 4 mg/day for 2 weeks). In the third week of the disease, he had had one episode of hemoptysis, and a repeat chest CT had revealed evidence of necrotizing pneumonia (Figure 1, B) with a widening of the cavitary lesion to about 30 mm. Therefore, he had received broad-spectrum antibiotics and the corticosteroid had been discontinued. The conditions of the patient had remarkably improved and he had been discharged at the end of the fourth week. 

On the current admission, his condition was stable, his blood pressure was 120/80 mmHg, his respiratory rate was 18/min, and his SpO_2_ in room air was 90%. His laboratory findings were anemia (Hb=11.7 gr/dL), mild leukocytosis (13200/mL, polymorphonuclear leukocytes=71.7%, lymphocyte=24.1%), and elevated inflammatory markers (erythrocyte sedimentation rate=34 mm/h, c-reactive protein=61 mg/L, lactic acid dehydrogenase=558 U/L). Results of other tests, including platelet count, coagulation test, liver, and renal function tests were within the normal range. It must also be mentioned that the SARS-CoV-2 polymerase chain reaction test was negative. 

Another chest CT scan showed two cavitary lesions in both of his lungs; the larger lesion measured 50×80 mm, was located at the site of the previous lesion with a thick wall, and contained soft dense material (Figure1, C and D). A suspicion of fungal infection was raised and the patient underwent a trans-thoracic biopsy of the cavitary lesion. Supposedly, the direct examination of the biopsy material revealed septal and branching hyphae, and a fungal culture grew *Aspergillus* species.

**Figure 2:**
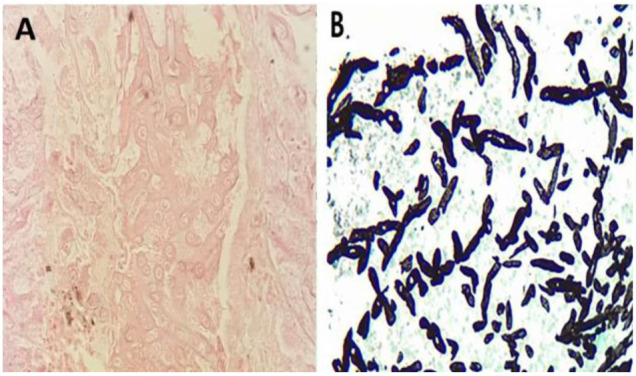
**A)** presence of markedly degenerated hyaline fungal elements in the necrotic background (100X, H&E staining)

Fungal DNA was extracted using a DNA extraction kit (Gene All DNA extraction kit; Gene All, Germany) according to the instructions of the manufacturer. The beta-tubulin region was sequenced to confirm the identification of *Aspergillus* species. The yielded sequence was subjected to the Basic Local Alignment Search Tool (BLAST) program (http://www.blast.ncbi. nlm.nih.gov/Blast). The DNA sequence of the beta-tubulin region matched that of *Aspergillus tubingensis* species, showing 99% similarity with the ex-type strain of the species. However, a histologic assessment revealed marked infiltration of a mixed population of inflammatory cells, including lymphoplasma cells, macrophages, and neutrophils (Figure 2, A). 

Moreover, large fragments of necrotic tissue containing degenerated forms of hyaline fungal elements were noted, which were more characterized in special Grocott's methenamine silver stain (Figure 2. B). The patient was treated with oral voriconazole (800 mg/day for 5 days followed by 400 mg/day). After starting the treatment, hemoptysis was stopped and a CT scan was performed two weeks later. The results showed that the bigger cavity was slightly larger while its wall was thinner and the mass was smaller. It was also found that the other cavity was changed into a small boll.

## Discussion

The COVID-19 is an emergent disease caused by a group of enveloped, single-stranded RNA viruses. The disease presents with different clinical manifestations and severity meaning that the presentation can be asymptomatic, mild, or even severe with high mortality. In many cases, the symptoms are so severe that they lead to death [ 8 ]. In about 20% of patients, the disease may lead to pneumonia that causes severe damage to the lungs and may warrant a need for intensive care. 

Patients with respiratory distress syndrome due to viral infections are prone to secondary infections. Accordingly, secondary fungal infections are increasingly being reported in patients with COVID-19 [ 9 , 10 ]. Since *Aspergillus* is an opportunistic pathogen, it can cause infection in these patients [ 4 ]. Aspergillosis has recently been observed in COVID-19 patients with severe pneumonia [ 4 , 11 , 12 ]. There are also numerous reports of patients with COVID-19 admitted to intensive care units exposed to risks of IPA [ 6 , 11 ]. 

The diagnosis of IPA requires careful examination since the respiratory symptoms, as well as the radiographic symptoms, can be similar to those seen in patients with COVID-19. Therefore, accurate sampling, good culture methods, and laboratory diagnosis are necessary to rule in or rule out aspergillosis [ 13 ]. Here we report a case of IPA in a patient with COVID-19. To our knowledge, there are limited studies that characterize black *Aspergillus* from patients with COVID-19. In particular, this is the first case report documenting the clinical and mycological characteristics of invasive aspergillosis caused by *A. tubingensis* in a patient with COVID-19 from Iran. 

In a study performed on 257 COVID-19 patients by Zhu et al., it was found that 243 patients had viral, bacterial, and fungal co-infections. More specifically, 60 (23.3%) patients had co-infection with fungal pathogens, including *Aspergillus* (n=60, 23.3%), *Mucor* (n=6, 2.5%), *Candida* (n=2, 0.8%), and *Cryptococcus* (n=1, 0.4%) [ 14 ]. Monique Freire Santana et al. reported IPA in a patient with COVID-19 [ 15 ]. The patient had comorbid conditions, including hypertension, type two diabetes mellitus, and chronic kidney disease that further weakened his immune status and made him prone to secondary fungal infections. 

Results of another study conducted by Deepak Bhonagiri et al. confirmed IPA by isolating *Aspergillus fumigatus* from the tracheal aspirate of a patient with severe COVID-19. Diagnostic discrimination between putative IPA and tracheal colonization might have been enhanced by testing the serum galactomannan [ 13 ]. Haglund et al. reported a COVID-19 patient with a positive history of percutaneous coronary intervention, type two diabetes, heart failure treatment with angiotensin-converting enzyme inhibitors, and obesity (body mass index: 36.8 kg/m^2^). The report also described that the galactomannan test was positive for the bronchoalveolar lavage sample of the patient, while the serum test was negative. They isolated *A. fumigates* from the patient and IPA was confirmed [ 16 ]. 

In another study carried out by Juergen Prattes et al., a COVID-19 male patient was reported with a medical history of chronic obstructive pulmonary disease GOLD grade two, obstructive sleep apnea syndrome, insulin-dependent type two diabetes with end-organ damage (retinopathy, nephropathy , and polyneuropathy), arterial hypertension, coronary heart disease, and obesity (body mass index: 38 kg/m^2^). Finally, the diagnosis of invasive aspergillosis was confirmed by the growth of *A. fumigatus* and a positive *Aspergillus*-Ag test in endotracheal aspirate [ 6 ]. 

Similar to the reported cases above, our case had a medical history, including hypertension and coronary artery bypass graft 3 years ago. With the confirmed diagnosis of COVID-19, the CT scan of the chest of the patient showed 40% involvement of the lungs, which indicated secondary infection by Aspergillus. The above-mentioned data and the results of the present study indicate that patients with COVID-19 can develop secondary fungal infections, such as aspergillosis that can occur as a result of their diminished immunity due to COVID-19 infection, comorbid medical conditions, and other reasons, such as their age and past medical history.

## Conclusion

In conclusion, patients with COVID-19, especially its severe form, are prone to multiple complications that may require hospitalization in the intensive care unit and cause a potential increase in mortality rate. Complications include a high risk for thrombosis and secondary bacterial and fungal infections, especially IPA as reported in our case. Patients with COVID-19 who are hospitalized in the intensive care unit for a long time are at the risk of developing IPA and increasing mortality as a result.

## Acknowledgement

The most sincere appreciation and thanks go to Mohammad Reza Safari, Pegah Afarinesh for their support and contribution. This study has been funded and supported by Tehran University of Medical Sciences (TUMS); Grant no.1400-1-99-51480.

## Authors’ contribution

M.R.S., N.K., and S.A.D.M conceived the study and treatment and also discussed the case and the implications. S.KH., A.I., E.S., R.S.K., Z.K., A.A., A.A.R., and M.A. diagnosed the case. S.KH., A.I., M.I.G, and M.R.S wrote the manuscript. All authors had full access to all data in the study and take responsibility for the integrity of the analysis.

## Financial disclosure

No financial interests related to the material of this manuscript have been declared.

## Conflict of Interest

The authors declare no conflict of interest related to this study.

## Ethical Considerations

This case report was performed in compliance with the Declaration of Helsinki and approved by the Ethics Committee of Tehran University of Medical Sciences, Tehran, Iran (IR.TUMS.VCR.REC.1399.152).
